# Molecular Investigation on a Triple Negative Breast Cancer Xenograft Model Exposed to Proton Beams

**DOI:** 10.3390/ijms21176337

**Published:** 2020-09-01

**Authors:** Francesco P. Cammarata, Giusi I. Forte, Giuseppe Broggi, Valentina Bravatà, Luigi Minafra, Pietro Pisciotta, Marco Calvaruso, Roberta Tringali, Barbara Tomasello, Filippo Torrisi, Giada Petringa, Giuseppe A. P. Cirrone, Giacomo Cuttone, Rosaria Acquaviva, Rosario Caltabiano, Giorgio Russo

**Affiliations:** 1 Institute of Molecular Bioimaging and Physiology (IBFM-CNR), 90015 Cefalù (Palermo), Italy; francesco.cammarata@ibfm.cnr.it (F.P.C.); giusi.forte@ibfm.cnr.it (G.I.F.); luigi.minafra@ibfm.cnr.it (L.M.); marco.calvaruso@ibfm.cnr.it (M.C.); giorgio.russo@ibfm.cnr.it (G.R.); 2 National Laboratory of South, National Institute for Nuclear Physics (LNS-INFN), 95123 Catania, Italy; pietro.pisciotta@lns.infn.it (P.P.); filippo.torrisi@unict.it (F.T.); giada.petringa@lns.infn.it (G.P.); pablo.cirrone@lns.infn.it (G.A.P.C.); cuttone@lns.infn.it (G.C.); 3 Department of Medical, Surgical and Advanced Technological Sciences “Gian Filippo Ingrassia”, Section of Anatomic Pathology, University of Catania, 95123 Catania, Italy; giuseppe.broggi@gmail.com (G.B.); rosario.caltabiano@unict.it (R.C.); 4 Department of Radiation Oncology, University Medical Center Groningen, 9713 Groningen, The Netherlands; 5 Department of Drug Science, Section of Biochemistry, University of Catania, 95125 Catania, Italy; roberta.tringali@live.it (R.T.); btomase@unict.it (B.T.); racquavi@unict.it (R.A.); 6 Department of Biomedical and Biotechnological Sciences (BIOMETEC), University of Catania, 95124 Catania, Italy

**Keywords:** triple-negative breast cancer (TNBC), proton therapy, xenograft mice, microarray

## Abstract

Specific breast cancer (BC) subtypes are associated with bad prognoses due to the absence of successful treatment plans. The triple-negative breast cancer (TNBC) subtype, with estrogen (ER), progesterone (PR) and human epidermal growth factor-2 (HER2) negative receptor status, is a clinical challenge for oncologists, because of its aggressiveness and the absence of effective therapies. In addition, proton therapy (PT) represents an effective treatment against both inaccessible area located or conventional radiotherapy (RT)-resistant cancers, becoming a promising therapeutic choice for TNBC. Our study aimed to analyze the in vivo molecular response to PT and its efficacy in a MDA-MB-231 TNBC xenograft model. TNBC xenograft models were irradiated with 2, 6 and 9 Gy of PT. Gene expression profile (GEP) analyses and immunohistochemical assay (IHC) were performed to highlight specific pathways and key molecules involved in cell response to the radiation. GEP analysis revealed in depth the molecular response to PT, showing a considerable immune response, cell cycle and stem cell process regulation. Only the dose of 9 Gy shifted the balance toward pro-death signaling as a dose escalation which can be easily performed using proton beams, which permit targeting tumors while avoiding damage to the surrounding healthy tissue.

## 1. Introduction

Breast cancer (BC) is a heterogeneous disease classified in several subgroups based on molecular and genomic profiles, associated with different treatment responses [1,2]. So, following a BC diagnosis, the most immediate challenge in patient management is the prognosis determination and identification of the most appropriate therapeutic approach. Moreover, specific BC subtypes are associated with a worse prognosis due to the absence of successful treatment plans. In this sense, the triple-negative breast cancer (TNBC) subtype, which represents 15–20% of BC incidence, is a clinical challenge for oncologists, because of its aggressiveness and absence of successful therapies. Considering that TNBC is featured by estrogen (ER), progesterone (PR) and human epidermal growth factor-2 (HER2) negative receptor status, patients cannot be treated with specific hormonal or targeted therapy, and a gold standard chemotherapy has not been established yet [3]. In addition, considering the molecular differences among BC subtypes, the choice of a unique treatment plan common to all BC patients, including radiotherapy (RT), may not be the best option. Moreover, taking into account a relapse rate ranging from 25% to 40% after treatment of TNBC, the acquisition of chemo and radioresistance is taken into consideration. However, several authors have demonstrated that breast conserving surgery in tandem with RT was less likely to develop locoregional recurrence compared with mastectomy [4,5,6].

Over the last decade, the technological development of RT has led to more performing and innovative technologies which can deliver, with high precision, increasing doses saving the organ at risk, and high-dose radiation on small-sized tumor targets [1,7,8]. This property of the proton beam is due to its typical curve of energy deposition through the matter, releasing the well-known Bragg peak, which can represent a better conformational option in respect to conventional photon beams [3].

Then, proton therapy (PT) provides a substantial physical advantage compared to conventional RT by using X or Gamma radiation rays, even if its biological advantages still remain understudied. 

However, in BC treatment, the prospective use of PT in place of conventional RT would result in a lower ionizing radiation (IR) dose to the heart and lungs, especially if the tumor is located in the left mammary gland [9,10,11,12,13,14]. Furthermore, several authors have debated the use of conventional RT for TNBC patients [11,15,16]. Because of such advantages and due to clinically encouraging results, PT is currently used for different aggressive forms of cancers and its use is rapidly growing, especially for those sited in the proximity of organs at risk. However, few data are available regarding proton-induced molecular changes, particularly in mammary gland breast cells, a topic that therefore deserves to be accurately described. 

In this scenario, we have recently described the dose response effects on cell survival induced by proton beam irradiation by using an in vitro approach, and, for the first time to our knowledge, the radiation-induced gene expression profiles (GEPs) and immunological profiles produced by the BC cell lines with different aggressive phenotypes including the well-known TNBC model, the MDA-MB-231 BC cells (metastatic, basal, triple-negative) [7]. 

Here we decided to generate a TNBC xenograft model using MDA-MB-231, with the aim to test the in vivo PT efficacy and to search for specific PT molecular signature, using increasing doses of proton beam (2, 6 and 9 Gy) [17]. The dose range of 2–9 Gy was chosen with the aim of understanding the different molecular response observable with the dose escalation. Furthermore, the dose of 2 and 9 Gy were precedingly used in our in vitro experiments, allowing us to perform a GEP analysis comparison between in vitro and in vivo molecular responses induced by the same PT dose. In addition, an immunohistochemical (IHC) characterization was performed to search for particular markers of specific biological processes. 

The results of this comparison show the activation of specific pathways associated with the dose delivered and the time window analyzed in the MDA-MB-231 xenograft model, which sustains the control of key cellular processes, such as stem cell proliferation, cell cycle and cell death, and is thus able to control cell fate. In particular, the major PT efficacy is evident with the dose of 9 Gy, in which the survival/cell death balance is clearly moved toward tumor cell killing. These findings encourage the use of proton beams for the treatment of more aggressive tumor forms because its specific energy deposition curve allows the use of dose escalation while saving organs at risk. 

## 2. Results

### 2.1. Monte Carlo Simulation Depth Dose Profile and LET (Linear Energy Transfer) Assessment

**Figure 1:**
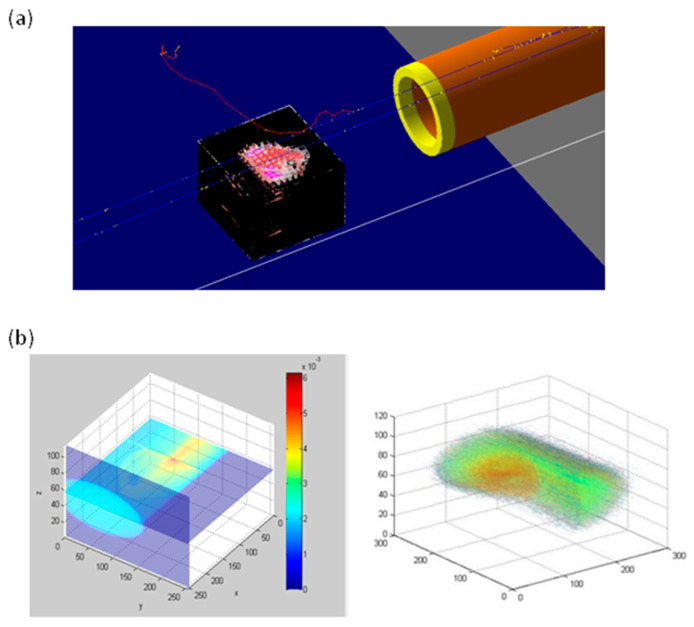
(**a**) Screenshot of DICOM volume in GEANT4. (**b**) Example of dose distribution calculated by Monte Carlo application.

As above described, specific treatment plans were developed for each animal used in this project. In turn, the best irradiation configuration and dose distributions were applied using the GEANT4-based application (GEometry ANd Tracking-4). DICOM (Digital Imaging and COmmunications in Medicine) CT images were employed to define the animal composition and geometry inside the GEANT4 framework. The medium LET (linear energy transfer) value calculated within the tumor was equal to 6.68 keV/μm. In Figure 1 an example of dose distribution calculated by our application is provided. 

### 2.2. Immunohistochemical Evaluation of Key Markers

Staining intensity of key regulator molecules involved in crucial cellular processes has been evaluated in order to better highlight the PT efficacy and understand which biological processes “are involved in the successful treatment plan” or “in the success of RT treatment” considering these results together with the whole genome GEP analysis, performed at the same doses. These markers’ expression has been correlated to the dose delivered (2, 6 e 9 Gy) in both the two-time window analyzed of 72 h and 10 days after PT. As shown in Table 1 these were: CD133 (Cluster of Differentiation 133) as cancer stem cells marker, Cyclin D1 as cell cycle positive regulator marker, ki67 as proliferation rate marker, cleaved Caspase 3 as apoptotic marker, CD68 (Cluster of Differentiation 68) as macrophage marker. In addition, Table 1 reports the evaluation of necrosis extension and the apoptotic cell count, in order to better elucidate the death mechanism induced by PT.

#### High Power Fields (HPF)

**Figure 2:**
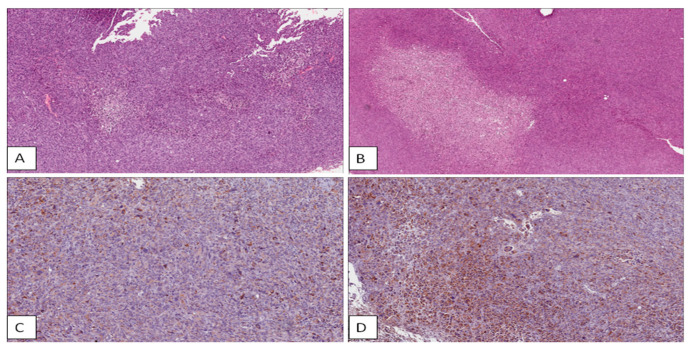
Representative slides of hematoxylin/eosin (H&E) and immunohistochemical staining. (**A**) Small necrosis foci after 10 days post-PT (proton therapy) with the dose of 9 Gy; (**B**) extensive necrosis after 72 h post-PT with the dose of 9 Gy; high immunohistochemical expression of caspase-3 (**C**) and CD133 (**D**) in tumor after 72 h post-PT with the dose of 9 Gy.

Figure 2A,B display representative H&E-stained slices deriving from samples analyzed 10 days and 72 h, respectively, after PT with 9 Gy of IR dose. In particular, after irradiation with 9 Gy, small foci of necrosis were observed 10 days post-irradiation (Figure 2A), while extensive necrosis was highlighted after 72 h (Figure 2B). In particular, the necrosis extension strongly increases with the dose escalation in the time window of 72 h post-PT, whereas 10 days after PT, we can observe an increase with the dose of 2 Gy with respect to untreated sample, and, then, a reduced necrotic extension at 6 and 9 Gy respect to the 2 Gy sample. Regarding apoptosis, a strong increase of cleaved CASP3 was highlighted in a dose- and time-dependent manners, except for samples treated with 9 Gy at the time window of 10 days post-PT. Figure 2C shows the high cleaved CASP3 expression after 72 h post-PT with the dose of 9 Gy.

In addition, the apoptotic count showed a slight increase with dose escalation in both the time window of 72 h and 10 days post-treatment. In order to understand the reduction of necrotic foci and cleaved CASP3 expression with higher doses in samples observed 10 days post-PT, we assessed the CD68, as a macrophage marker. Indeed, its positivity was found only in samples analyzed 10 days after PT, with higher scores just in the 9 Gy treated sample, rendering evident the scavenger effect by these cells, able to eliminate dead tumor cells and debris.

On the other hand, survival signals are activated by the treatment, as suggested by a mild increase of Cyclin D1 in samples treated with all the three doses, both after 72 h or 10 days from PT exposures, with the only exception of the 9 Gy sample analyzed after 10 days, most likely due to the above-mentioned scavenger effect by macrophages. Finally, the stem cell marker CD133 was found up-regulated after PT using all the doses of 2, 6 and 9 Gy in the early time-window of 72 h. Instead, after 10 days post-irradiation, a CD133 strong increase is observed with respect to the untreated sample, and then a progressive reduction with 6 and 9 Gy in respect to the 2 Gy sample. Figure 2D shows the CD133 expression in a tumor treated with 9 Gy observed 72 h post-PT. Finally, the Ki67 marker expression did not show significant changes. 

### 2.3. Overview of cDNA Microarray Gene Expression Analyses

As above described, in this work we analyzed the GEPs induced by PT irradiation using 2, 6 and 9 Gy of IR doses, on BC xenograft mice models in order to highlight genes and cellular processes involved in radioresistance/radiosensitivity balance regulation.

In detail, we analyzed the three following configurations:

(i) MDA-MB-231 xenograft mice_2Gy_PT
(ii) MDA-MB-231 xenograft mice_6Gy_PT
(iii) MDA-MB-231 xenograft mice_9Gy_PT

Comparative differential gene expression analyses revealed that a conspicuous number of genes had significantly altered expression levels by 2-fold or greater, compared to the untreated reference group as follows: MDA-MB-231 xenograft mice_2Gy_PT, 1256 differentially expressed genes (DEGs; 457 down-regulated and 799 up-regulated); MDA-MB-231 xenograft mice_6Gy_PT, 848 DEGs (279 down-regulated and 569 up-regulated); and MDA-MB-231 xenograft mice_9Gy_PT, 1279 DEGs (407 down-regulated and 872-up regulated).

Moreover, up- and down-regulated transcripts for each configuration analyzed in this study were selected and grouped according to their involvement in specific biological pathways using the DAVID tool, as displayed in Table 2, Table 3, Table 4 and Table 5.

In detail, as shown in Table 2, Table 3 and Table 4, graft-versus-host disease, allograft rejection and phagosome cellular pathways were deregulated in all the three configuration assayed in this work, underling their key role strictly linked to proton irradiation rather than to the dose delivered. 

The first two pathways are known to be related to immune reactivity of the recipient against the transplanted allograft. On the other hand, a phagosome is able to maintain host homeostasis because it is involved in the degradation of pathogens and cellular death as well as in other processes linked to antigen-presenting processes and the recovery of inflammatory mediators [18].

In addition, after 2 Gy of PT, our preclinical tumor model was able to deregulate the antigen processing and presentation pathway, once again underlying the immune system activation after PT, as well as the cell adhesion molecules (CAMs) involved in binding and communication with other cells.

On the other hand, after 6 Gy of PT irradiation, leukocyte transendothelial migration process and proteoglycans in the cancer pathway were deregulated. Precisely, leukocyte migration from the blood into tissues is vital for immune surveillance and inflammation while proteoglycans are key molecules and effectors of cell surface and microenvironments, known to have multiple functions in cancer and angiogenesis due their ability to regulate neoplastic growth and neovascularization [19].

Interestingly, after 2 and 6 Gy of PT irradiation, we selected only the top five statistical and biological relevant pathways. In contrast, after 9 Gy of PT irradiation we selected more numerous deregulated cellular networks and, thus, in Table 4 we reported the top 15 statistical and biological relevant pathways. In summary, some of these are involved in cell–cell communication and/or immune system activation (i.e., antigen processing and presentation; Rap1 signaling pathway; graft-versus-host disease; chemokine signaling pathway; focal adhesion; allograft rejection, platelet activation, phagosome), others are related to tumor progression, angiogenesis and invasiveness (i.e., pathways in cancer; VEGF signaling pathway, proteoglycans in cancer; Ras signaling pathway, signaling pathways regulating pluripotency of stem cells; HIF-1 signaling pathway; Wnt signaling pathway). Candidate genes were selected and used to validate microarray datasets by qRT-PCR analyses that confirm gene-expression trends (Table 5). Interestingly, as displayed, MDA-MB-231 xenograft mice exposed to PT with 2 and 6 Gy have shown overall comparable gene expression trends, unlike samples treated with the high dose of 9 Gy. 

### 2.4. Commonly Deregulated Genes and Pathways among the PT Schedules

**Figure 3:**
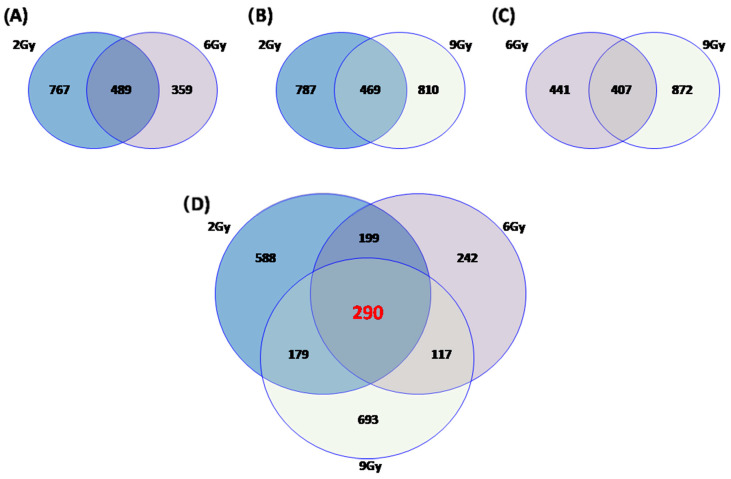
Venn diagram of the number of unique and shared differentially expressed genes after proton irradiation in MDA-MB-231 xenograft mice using 2, 6 and 9 Gy of radiation doses as follow: (**A**) unique and shared differentially expressed genes in samples treated with 2 and 6 Gy; (**B**) unique and shared differentially expressed genes in samples treated with 2 and 9 Gy; (**C**) unique and shared differentially expressed genes in samples treated with 6 and 9 Gy; (**D**) unique and shared differentially expressed genes in samples treated with 2, 6 and 9 Gy.

Moreover, in order to study the number of unique and shared differentially expressed genes between MDA-MB-231 xenograft mice exposed to IR doses of 2, 6 and 9 Gy, we performed Venn diagrams (Figure 3). As shown, a great amount of genes were commonly deregulated in the configurations assayed: 489 genes were deregulated after 2 and 6 Gy of proton irradiation; 469 genes changed their expression levels after 2 and 9 Gy; and 407 were deregulated both after 6 and 9 Gy of PT, respectively shown in Figure 3A–C. Finally, as shown in Figure 3D, we decided to highlight the deregulated gene set shared between all the configurations assayed (2, 6 and 9 Gy) and, thus, we selected the 290-gene signature linked to proton cell response rather than to the dose delivered (Figure 3D). The respective DAVID analysis of this gene list results in the following pathways: antigen processing and presentation; graft-versus-host disease; allograft rejection; phagosome and complement and coagulation cascades signaling (Table 6), confirming once again the crucial role of immune system activation, in response to irradiation by proton beams of a xenograft model.

## 3. Discussion

One of the main aims of our research group is to fill the gap regarding the radiobiological knowledge of the molecular responses induced by PT and its potential advantages for treating radioresistant tumor subtypes. In addition, several authors have reported controversies with respect to the use of conventional RT for patients with TNBC (ER–/PR–/HER2–) [15,16]. In this sense, the absence of hormonal or targeted therapy against TNBC makes it a clinical challenge for oncologists in terms of patient management. 

According to these assumptions, we decided to investigate the molecular responses induced by PT in MDA-MB-231 BC cells, chosen as the best model of a TNBC disease, by using in vitro and in vivo approaches, in order to clarify mechanisms involved in the treatment success. This knowledge could help us to identify possible targets to induce radio-sensibilization and shift the balance of cell fate towards death. Furthermore, we aimed to use protons because they offer the physical advantage of reaching the target site while saving the organs at risk, such as heart and lungs. In addition, as suggested by some authors, a variable instead of fixed RBE (Relative Biological Effectiveness) (1.1) should be considered along the proton energy deposition curve (Bragg curve), taking into account the RBE dependency on linear energy transfer (LET) and tissue properties (α/β values) [20,21]. Particularly, there is an inverted relationship between proton energy and LET, corresponding to an RBE increase especially in the distal SOBP (Spread-out Bragg peak). These properties make them more suitable for those radioresistant tumors. 

The dose range of 2–9 Gy has been chosen with the aim of understanding the different molecular response observable with the dose escalation. In particular, the doses of 2 and 9 Gy were previously used in our in vitro experiments, both using photons/electrons or protons, allowing us to use the possibility to make a GEP analysis comparison. It has to be remembered that 2 Gy is the daily dose of a conventional RT fractionated treatment, whereas 9 Gy is used as a boost dose in the IOERT (IntraOperative Electron Radiation Therapy) technique. 

In particular, we highlighted a strong activation of the immune response in MDA-MB-231 cells subjected to PT irradiation in a time-dependent manner [7]. Indeed, MDA-MB-231 cells showed the strongest potentially pro-inflammatory profile compared with other BC cell lines analyzed. The activation of intracellular inflammatory-related pathways justifies the MDA-MB-231 cells’ capacity to release inflammatory molecules in tumor microenvironment, then driving cell fate balance, senescence mechanism, angiogenesis and cell migration. Taken together, these data strongly suggest that the TNBC cells’ aggressive phenotype and their resistance to treatments are sustained by the activation of intracellular pro-inflammatory mechanisms, which are known to be a typical response to stress stimuli such as irradiation. Notably, this particular behavior is peculiar to MDA-MB-231 and it is not shared by other cell lines studied previously by our group, as for example in MCF7 cells, which had a very low activation of the immune response, both for quantity and types of released cytokines, and, instead, showed a molecular response to irradiation driven by the p53 pathway [7,8,22]. 

Then, here we used an immunohistochemical approach and a whole-genome cDNA microarray to better understand the real in vivo response to PT irradiation, in respect to what we previously observed in vitro, applying the same doses. 

Table 1 displays the quantification of some key biomarkers involved in cell fate balance after irradiation, by IHC approach. These biomarkers are crucial actors in cell cycle regulation, stem cell regenerative capability or death processes, such as apoptosis or necrosis; for the latter the percentage extension was also determined.

Table 2, Table 3, Table 4 and Table 6 display the top 5–15 significant pathways activated in the tumor tissues of our xenograft models in response to 2, 6 and 9 Gy, respectively, using untreated tumor tissues as control, resulting in a relative GEP quantification. It can be observed that the three dose configuration used (2, 6 and 9 Gy) share: (1) a strong cancer cell communication, driven by CAM molecules; (2) graft-versus-host disease due to the xenografts model creation; (3) the activation of stem cells pathway known to be involved in radioresistant phenotype; (4) an overall up-regulation of inflammation biomarkers confirming, once again, the MDA-MB-231 great inflammatory potential.

In comparison with our previous data on the MDA-MB-231 in vitro model, the first two above mentioned pathways are peculiar of the PT response by the animal model, due to the presence of a tridimensional tissue in this case, which amplify the cell–cell communication, and due to the insertion of a human tumor cell line into the mouse model, which activates the well-known host versus graft response, here observable despite the fact that we used nude immunosuppressed mice.

On the other hand, the other two pathways (3 and 4) are common in the in vitro and in vivo models, confirming the PT capacity, even at low doses, to activate a certain tumor counterattack strategy for radioresistance, using inflammation and stem cell activation, which try to direct cell fate towards survival and proliferation.

In this regard, even the literature describes the role of remaining tumor cancer stem cells before the RT starting and their ability to repopulate over the course of treatment plan in several conditions including hypoxia, stroma interaction among cells and variations in the intrinsic cells’ sensitivity to radiation, as well as in the modulation of DNA repair or other cell survival pathways [23,24,25,26,27]. Thus, we better explored the stem cell marker modulation in our BC xenograft model during PRT plans, considering our GEP and IHC data (Table 1 and Table 5). In particular, an up-regulation of CD24 and CD44 gene expression was assessed both by microarray technology and by qRT-PCR in all the three dose configurations assayed at 72 h post-RT. Similarly, using IHC, we reported the CD133 up-regulation after PT using all the doses 2, 6 and 9 Gy in the early time-window of 72 h. Instead, after 10 days post irradiation, a CD133 progressive reduction is observed, in respect to untreated mice, making us hypothesize that the tumor stem potential downregulation is a late effect of PRT. This opposite trend, between the two-time windows analyzed, is probably due to the scavenger process driven by macrophages, which is more evident after more days. Indeed, the macrophage CD68 marker was positive only in the 10 days post-RT samples, by IHC approach (Table 1), confirming their role in tissue remodeling during the days after PT.

We also investigated the cell cycle modulation after PT, observing the CDC20, CDC25 and CCNA decreased gene expression after the administration of the dose of 2 Gy (Table 5), and their upregulation 72 h post-PT with 9 Gy. However, a mild increase of Cyclin D1 is observed, by IHC approach, in samples treated with all three doses, both after 72 h or 10 days from PT exposure, with the only exception of the 9 Gy sample analyzed after 10 days, most likely due to the above-mentioned scavenger effect by macrophages. 

These results make evident the role of irradiation in stimulating cell cycle progression and stem cell proliferation in MDA-MB-231 cells, thus activating radioresistance, especially observable at low doses in early post-treatment time windows.

Besides, we assayed the Ki67 immunomarker, a nuclear protein that is expressed exclusively during the active cell cycle phases, but not in resting cells. As known, Ki67 is widely used in pathology to assess cell proliferation within multiple different neoplasms including BC [28]. In BC, Ki67 has shown a promising role as an independent prognostic marker and as a predictive marker of responsiveness or resistance to therapies, with a consolidated prognostic utility [29]. In our experiment, no statistical variation among the PT configurations assayed was reported, as displayed in Table 1. 

On the other hand, in order to analyze the activation of the cell death process after PT exposure, we assayed the amount of the main apoptosis key regulator by IHC: the CASP3 cleaved protein. As reported in Table 1 a strong increase of CASP3 was highlighted in a dose- and time-dependent manner, except for samples treated with 9 Gy at the time window of 10 days post-PT. Similarly to what we observed for the CD133 and Cyclin D1 biomarkers, this observation could be the result of scavenger action, which removed the dead cells. In line with the above-reported cell cycle and apoptosis trends, a BAX down-regulation was reported after 2 Gy, and, conversely, an up-regulation after 9 Gy of IR dose at 72 h post-PT (Table 5). In addition, considering the apoptotic count, assessed by morphology, a slight increase is observed with increasing dose in both samples analyzed at 72 h and 10 days post-treatment. 

As well described in the literature, IR activates complex cross-linked intracellular networks, able to define cell fate in the choice between survival and death. It has become evident that, in particular for solid tumors, the inhibition of neoplastic cell proliferative capacity following irradiation can occur through different types of cell death (i.e., apoptosis, necrosis, mitotic catastrophe, autophagy and senescence) [30]. However, necrosis has generally been considered as a predominant cell death process after the administration of treatment with high IR doses, while at a lower doses, it has been recognized as a passive and unregulated event [31]. Then, we also evaluated the number of necrotic foci and the percentage of necrosis extension by morphology, as displayed in Table 1. 

In the early time window of 72 h post-PT, a strong increase of necrotic extension is recognizable with the dose increase, whereas 10 days after PT, we can observe a strong increase with the dose of 2 Gy in respect to untreated sample, and a reduced necrotic extension using 6 and 9 Gy in respect to the 2 Gy sample. Again, this negative trend reported in the late time window of 10 days post-PT makes evident the scavenger activity to clear cellular debris, promoting phagocytosis, and mediating the recruitment and activation of other macrophages. Indeed, the CD68 positivity correlates with the dose delivered: 5–20% after 2 and 6 Gy; 20–50% after 9 Gy of IR doses, respectively. 

A tumor size reduction post-PT was not revealed by digital caliper, since it would have been necessary to wait for longer than 10 days, during which tumors treated with low doses would have grown beyond the maximum limit imposed for the animal sacrifice, in order to avoid suffering. Therefore, the in-depth study of the above described biological processes has become necessary to clarify the PT effect in our xenograft TNBC models.

Among the early activated GEP within 72 h post-PT, we confirm the activation of a key transcription factor (TF) as FOS, known to be related to the response to radiation, but poorly described following unconventional treatment modalities, as after PT. TFs regulate a wide spectrum of genes involved in inflammation, apoptosis, invasion and angiogenesis processes, contributing to confer tumor cell radioresistance [30,32,33]. In particular, AP-1 proteins (assembled from JUN and FOS proteins), and, above all, c-Fos, play an important role in the induction and development of radiation late effects in normal tissues. The JunB gene is responsive to IR and is immediately induced early after stimulation [30].

Finally, the DAVID analysis of commonly deregulated genes and pathways, among the PT schedules, confirms the involvement of some immunological processes, described above as strictly linked to proton cell response in MDA-MB-231 xenograft model: antigen processing and presentation; graft-versus-host disease; allograft rejection; phagosome and complement and coagulation cascades signaling (Table 6).

Moreover, we also confirm the activation of pathways poorly described in the literature, but previously observed by our group, such as the Rap1 signaling pathway and the Phagosome activation [34]. Rap1 gene encodes a protein that is part of a complex involved in the regulation of telomere length, possibly involved in the senescence process activation [35]. The regulation of phagosomes could be involved in radiation-induced autophagy, known to be able to enhance radioresistance and leading to activation of the survival pathway, as recently observed [36].

## 4. Material and Methods

### 4.1. Dose Evaluation and Distribution by Monte Carlo GEANT4 Toolkit

In order to optimize the irradiation procedures and dosimetry for in vivo experiments we decided to use a Monte Carlo approach using GEANT4 toolkit, as previously described by our group [8,21]. All proton treatments were performed at the INFN-LNS CATANA proton therapy facility in Catania (Italy) using a passive fixed horizontal proton beam line with an energy of 62 MeV/A. A beam shaping system was used to obtain a uniform dose distribution at the isocenter [21,37,38]. To optimize animal irradiation schedules and to obtain a precise and reproducible irradiation setting, we developed a dedicated positioning animal holder system [21,37,39]. All animals were irradiated using a degraded spread-out Bragg peak (SOBP), thanks to the use of a poly (methyl methacrylate) (PMMA) modulator wheel. The dosimetry was performed using a Markus ionization chamber (PTW Freiburg GmbH, Germany) and gafchromic EBT3 films (ISP Corp., New York, NY, USA) with an accuracy better than 3%. According to irradiation procedures conventionally used during PT in clinical practice, the dose delivery was monitored by a transmission ionization chamber placed along the beam line. All animal irradiations were performed at the same time interval of the day to guarantee no difference related to mice circadian rhythm, with a constant dose rate of 5 Gy/min.

Secondly, in order to perform an accurate and efficient prediction of the dose distribution inside tumors, a Monte Carlo simulation was applied. Moreover, the GEANT4 Monte Carlo toolkit was used to reduce irradiation of organs at risk (OAR). In turn, a previously developed and validated GEANT4-based application was used to perform a preliminary assessment of dose map, using the real mice anatomical structures. Thus, we evaluated linear energy transfer (LET) distribution [21] defining, voxel-by-voxel, the real target composition thanks to the use of a preclinical micro-PET/CT and DICOM micro-CT images (Albira Si, Bruker, Belgium), available at CAPiR (Centre for Advanced Preclinical in vivo Research), University of Catania, Italy [21]. The datasets were acquired using 600 views in high-resolution configuration, X-Ray energy of 45 kVp, a current of 400 µA and the dimension of each CT-voxel was equal to 125 × 125 × 125 µm^3^. 

### 4.2. Animal Model

#### Ethics Statement and Animal Model

The experiments were performed in accordance with the European Communities Council directive and Italian regulations (EEC Council 2010/63/EU and Italian D.Lgs. 26/2014). The project was approved by the Italian Ministry of Health (authorization number n. 527/2016-PR, approved on 26 May 2016). Efforts were employed to replace, reduce and refine the use of laboratory animals. To avoid irrelevant suffering to treated mice, euthanasia was performed as soon as the final score was reached. The endpoint used to determine if animals should undergo euthanasia was reached when tumor lesions showed a dimension higher than 1.2 cm and/or weight loss more than 20%.

All reasonable efforts were made to ameliorate suffering, avoiding the most painful procedures. To minimize suffering and mice distress, standard environmental enrichment of two nestles, a cardboard Fun Tunnel and one wooden chew block were provided.

Experiments were performed on 8 weeks old BALB/c Nude female mice (Charles River Laboratory), weighing 24 ± 3 g. Animals were housed in IVC cages at constant temperature (23–25 °C) under a 12/12 h light/dark cycle with ad libitum access to food and water. Mice were housed using a stocking density of 3 mice per cage in individual IVC cages. 

A total of 4 × 10^6^ MDA-MB-231 BC cells were inoculated in a total number of 24 mice (18 animals to treat with PT and 6 controls) [7,22,34]. Animal health and behavior were monitored twice a week together with body weight and clinical specific signs up to the sacrifice. After two weeks of growth the tumors reached the size of 8 +/− 2 mm, monitored by digital caliper, and irradiation treatments were performed. Proton irradiations were executed with doses of 2 Gy (6 mice), 6 Gy (6 mice) and 9 Gy (6 mice). So, 72 h and 10 days post-PT treatments, tumors were measured again using a digital caliper, then mice were sacrificed and treated tumors (as well as untreated ones used as control) were collected and stored at −80 °C until molecular analyses. 

### 4.3. Immunohistochemistry (IHC)

Tumor sections from MDA-MB-231 xenografts in Balb/c nude mice were collected at specific time points (72 h and 10 days) after PT treatments using 2, 6 and 9 Gy of IR doses. Sections were processed as previously described [40]. Briefly, the slides were dewaxed in xylene, hydrated using graded ethanol and incubated for 30 min in 0.3% H_2_O_2_/methanol to quench endogenous peroxidase activity, then rinsed for 20 min with phosphate-buffered saline (PBS; Bio-Optica, Milan, Italy). The sections were heated (5 min × 3) in capped polypropylene slide-holders with citrate buffer (10 mM citric acid, 0.05% Tween 20, pH 6.0; Bio-Optica, Milan, Italy), using a microwave oven (750 W) to unmask antigenic sites. To reduce the commonly seen non-specific immunoreactivity due to endogenous biotin, sections were pretreated with 10 mg/mL of ovalbumin in PBS followed by 0.2% biotin in PBS, each for 15 min at room temperature. Then, the sections were incubated with rabbit monoclonal anti-Cyclin D1 antibody (sp4; Diagnostics Biosystems, Pleasanton, CA, USA), diluted 1:50 in PBS (Sigma, Milan, Italy); rabbit monoclonal anti-estrogen receptors (ERs) antibody (ep1; DAKO, Glostrup, Germany), diluted 1:50 in PBS (Sigma, Milan, Italy); rabbit monoclonal anti-progesterone receptors (PgRs) (PgR636; DAKO, Glostrup, Germany) diluted 1:50 in PBS (Sigma, Milan, Italy); mouse monoclonal anti-Ki-67 antibody (MIB-1; DAKO, Glostrup, Germany), diluted 1:100 in PBS (Sigma, Milan, Italy); rabbit polyclonal anti-c-erb-B2 oncoprotein antibody (DAKO, Glostrup, Germany), diluted 1:500 in PBS (Sigma, Milan, Italy); mouse monoclonal anti-CD68 antibody (pg-m1; DAKO, Glostrup, Germany), diluted 1:100 in PBS (Sigma, Milan, Italy); rabbit polyclonal anti-CD133 antibody (Abcam, Cambridge, UK), diluted 1:200 in PBS (Sigma, Milan, Italy); rabbit polyclonal anti-cleaved caspase-3 antibody (Abcam, Cambridge, UK), diluted 1:50 in PBS (Sigma, Milan, Italy). The secondary biotinylated anti-mouse antibody was applied for 30 min at room temperature, followed by the avidin-biotin–peroxidase complex (Vector Laboratories, Burlingame, CA, USA) for a further 30 min at room temperature. The immunoreaction was visualized by incubating the sections for 4 min in a 0.1% 3,3′-diaminobenzidine (DAB) and 0.02% hydrogen peroxide solution (DAB substrate kit, Vector Laboratories, CA, USA). The sections were lightly counterstained with Mayer’s hematoxylin (Histolab Products AB, Göteborg, Sweden) mounted in GVA mountant (Zymed Laboratories, San Francisco, CA, USA) and observed with a Zeiss Axioplan light microscope (Carl Zeiss, Oberkochen, Germany).

Staining intensity score (IS) was obtained by two independent pathologists (RC, GB) and graded on a 0–3 scale, according to the following assessments: no detectable staining = 0, weak staining = 1, moderate staining = 2, strong staining = 3. Moreover, the percentage of immunopositive cells (Extent Score, ES) was scored in five categories: <5% (0); 5–30% (+); 31–50% (++); 51–75% (+++), and >75% (++++). Counting was performed at 200× magnification. In addition, staining intensity was multiplied by the percentage of positive cells to obtain the intensity reactivity score (IRS); IRS < 6 was considered as low expression (L-IRS), IRS > 6 was considered as high expression (H-IRS). The percentage of intratumoral CD68+ histiocytes was assessed according to the guidelines for evaluating the tumor-infiltrating lymphocytes (TILs) in breast cancer [41]. 

Only CD68 + histiocytes within the tumor’s borders were evaluated. CD68 + cells outside of the tumor borders were not included. Necrotic areas were excluded from the evaluation. CD68 percentage was reported as the average of histiocytes in the stromal component of the tumor. Thus, 3 groups were identified: 0–10% stromal CD68 + cells (group 1); 20–40% (group 2); 50–90% (group 3). 

In addition, as shown, the dosage and time of death, the number of necrotic foci, the necrosis extension (in terms of percentage of the total tumor tissue, %), were also reported in Table 1 for all the configurations assayed in this work. 

### 4.4. Apoptotic Count

The apoptotic count was performed by two pathologists (RC; GB) on hematoxylin and eosin-stained sections. The apoptotic count was assessed as the total number of apoptotic cells per 10 high power fields (HPFs) at 40× magnification using a Zeiss Axioplan light microscope (Carl Zeiss, Oberkochen, Germany).

### 4.5. Whole Genome cDNA Microarray Expression Analysis

In this work, we performed whole-genome cDNA microarray gene expression analyses, to study the biological processes activated in MDA-MB-231 xenograft mice models following 72 h post-PT, highlighting specific key genes involved in cell response to radiation and to select potential new biomarkers of radiosensitivity and radioresistance as previously described [7,42,43]. Then, 72 h post-PT treatments, mice were sacrificed; treated and untreated (used as a control) tumor tissues, were collected and stored until processing of molecular analyses. cDNA Microsoft was performed as previously described. Total RNA was extracted from cells using Trizol and the RNeasy mini kit (Invitrogen) and RNA concentration and purity were determined using a Nanodrop ND-1000 (Thermo Scientific Open Biosystems, Lafayette, CO, USA). Samples with an RNA integrity number (RIN) value of 10, assessed by using a Bioanalyzer 2100 (Agilent Technologies, Santa Clara, CA, USA), were used for further microarray analyses. Thus, according to the Agilent Two-Color Microarray-Based Gene Expression Analysis protocol, we studied the GEPs induced by 2, 6 and 9 Gy of PRT in MDA-MB-231 xenograft mice models. So, cRNA synthesis, labelling with Cy dyes, hybridization onto Whole Human Genome 4 × 44 K microarray GeneChips (Agilent Technologies) and microarray image detections, were conducted as reported [44]. Finally, statistical data analysis, background correction and normalization of the GEPs were performed using Feature Extraction and GeneSpring GX 13.0 softwares (Agilent Technologies). Genes were identified as being differentially expressed if they showed a fold change (FC) of at least 2 with a *p*-value < 0.05 compared with radiation untreated tumor, used as reference. GEP data included in this paper are available in compliance with Minimum Information About a Microarray Experiment (MIAME) standards, using the following Gene Expression Omnibus (GEO) accession number: GSE149023.

Finally, we studied biological pathways regulated by the genes belonging to the differentially expressed gene lists obtained by GEP analyses, firstly using the Database for Annotation, Visualization and Integrated Discovery (DAVID) network building tool (https://david.ncifcrf.gov/tools.jsp), that provides a comprehensive set of functional annotation for investigators to study the biological content captured by high throughput technologies such as microarray analyses and secondly by using PubMatrix tool in order to confirm our assumptions [45].

### 4.6. qRT-PCR Analysis

Candidate genes for qRTPCR analysis were chosen based on the microarray results. Total RNA was reverse-transcribed into cDNA and then analyzed by real-time PCR in triplicate using a Fast 7500 Real-Time PCR System (Applied Biosystems, Carlsbad, CA, USA), as previously described [46]. The oligonucleotide primers chosen for qRT-PCR were selected with Primer3 software and tested as previously described [46]. Quantitative data, normalized versus that for the rRNA *18S* gene, were generated from three independent experiments and analyzed by the average of triplicate cycle threshold (Ct) according to the 2^−ΔΔct^ method using SDS software (version 1.4, Applied Biosystems, Carlsbad, CA, USA). The data shown and the values are expressed as the mean ± SD relative to mRNA levels in the untreated MDA-MB-231 xenograft mice not exposed to PRT, used as the control sample.

## 5. Conclusions 

As described above, the main aim of this work was to analyze the in vivo molecular response to PT and its efficacy in a MDA-MB-231 TNBC xenograft model, also describing for the first time to our knowledge specific PT molecular signatures linked to the dose delivered. 

Summarizing, our study reveals the effect of single shots of PT doses on a TNBC xenograft model, in the two-time window of 72 h and 10 days post-PT, showing a detailed molecular response for each configuration studied. In particular, the GEP analysis reveals a great immune response activation by the tumor tissue itself, due to irradiation, as well as a tentative tumor radioresistance through the positive modulation of cell cycle and stem cell process. 

**Figure 4:**
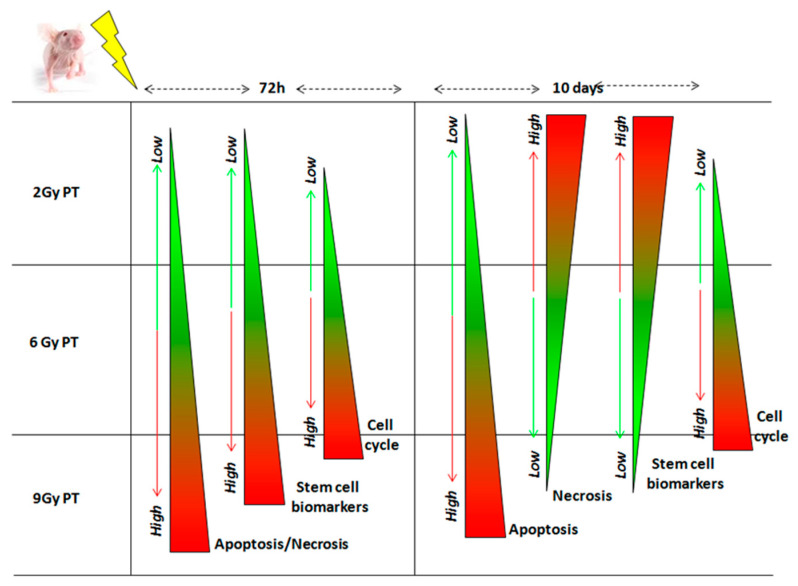
The figure displays the main remarks of this work. Green and red color represent the down- and up-regulation of the selected process after PT exposures, respectively. Stem cell activation: after 72 h post-PT with 2, 6 and 9 Gy, we assessed the up-regulation of CD24 and CD44 genes and by CD133 up-regulation. After 10 days post-irradiation, a CD133 progressive reduction was observed. Cell cycle modulation: a decreased gene expression followed by an up-regulation of CDC20, CDC25 and CCNA gene expression was measured according to the increasing dose delivered after 72 h post-PT. In addition, a mild increase of Cyclin D1 is observed, both after 72 h or 10 days from PT exposures. Apoptosis activation: a strong increase of CASP3 was highlighted in a dose- and time-dependent manners, except for samples treated with 9 Gy at the time window of 10 days post-PT. Moreover, the apoptotic count reported a slight increase according to the increasing dose, in both samples analyzed at 72 h and 10 days post-PT. Necrosis: after 72 h post-PT a strong increase of necrotic extension is recognizable with the dose increase, whereas 10 days after PT, a strong increase with the dose of 2 Gy in respect to untreated sample, and a reduced necrotic extension using 6 and 9 Gy in respect to the 2 Gy sample, were reported.

Only the dose of 9 Gy showed evident effects of moving balance toward tumor cell death. Further evidence is the role of both innate immunity and macrophage cells which were actively attracted within the tumor to remove tumor dead cells and debris, a process evident even in our cell models of nude mice. Figure 4 displays in a synoptic way the conclusive remarks of this study, describing the trend of main cellular processes involved in the balance between death and survival, with respect to the increase of delivered dose (an activation of stem cell pathway; a modulation of cell cycle process; the apoptosis regulation and a strong increase of necrotic extension recognizable with the dose increase). 

As suggested by some authors, the proton energy deposition along the Bragg curve permits evaluation of a variable instead of fixed RBE, especially higher in the distal SOBP. These properties make protons more suitable to deliver higher total doses and dose per fractions, with dedicated treatment plans for more radioresistant tumors, targeted with much higher precision while saving surrounding healthy tissues and organs at risk [20,21]. 

## Figures and Tables

**Table 1:** Immunohistochemical tumor characterization.

Samples	Number of Necrotic Foci	Extension of Necrosis (%)	Caspase-3			CD133			Ki67	CD68	Cyclin D1	Apoptotic Count (10 High Power Fields)
			IS	ES	IRS	IS	ES	IRS				
Untreated (72 h)	0	0	1	1	1	2	1	2	60%	Negative	Negative	10
Untreated (10 days)	4	0.45	2	4	8	3	3	9	70%	Negative	Negative	11
MDA-MB-231 Xenograft mice exposed to 2 Gy (72 h)	1	10%	1	2	2	2	2	4	50%	Negative	Positive (+IS 2; ES 2; IRS 4)	12
MDA-MB-231 Xenograft mice exposed to 2 Gy (10 days)	3	20%	2	3	6	3	3	9	65%	Positive (+5–20%)	Positive (+IS 1; ES 2; IRS 2)	16
MDA-MB-231 Xenograft mice exposed to 6 Gy (72 h)	3	25%	2	4	8	3	3	9	50%	Negative	Positive (+IS 2; ES 2; IRS 4)	14
MDA-MB-231 Xenograft mice exposed to 6 Gy (10 days)	2	15%	2	2	4	2	3	6	70%	Positive (+5–20%)	Positive (+IS 2; ES 2; IRS 4)	14
MDA-MB-231 Xenograft mice exposed to 9 Gy (72 h)	2	50%	2	4	8	3	3	9	60%	Negative	Positive (+IS 2; ES 3; IRS 6)	15
MDA-MB-231 Xenograft mice exposed to 9 Gy (10 days)	1	5%	1	2	2	2	2	4	60%	Positive (++20–50%)	Positive (+IS 1; ES 3; IRS 3)	14

IS: intensity score (0–3); ES: extent score (0–4); IRS: intensity reactivity score (0–12). CD68 positivity was expressed using (+) symbol scale: lower positivity was expressed with one (+), while its increment with multiple (+).

**Table 2:** GEPs (gene expression profiles) DAVID analysis of MDA-MB-231 xenograft mice exposed to 2 Gy of proton irradiation.

Top 5 Molecular Pathways of Differentially Expressed Genes of MDA-MB-231 Xenograft Mice Exposed to 2 Gy of Proton Irradiations				
	Term	Count	*p* Value	Genes
1	Graft-versus-host disease	12	8.1 × 10^−7^	*HLA-DQB1, HLA-DRB1, HLA-A, IL1B, FASLG, HLA-C, HLA-DPA1, HLA-B, HLA-DPB1, HLA-DOA, HLA-E, HLA-DRA*
2	Allograft rejection	11	2.1 × 10^−5^	*HLA-DQB1, HLA-DRB1, HLA-A, FASLG, HLA-C, HLA-DPA1, HLA-B, HLA-DPB1, HLA-DOA, HLA-E, HLA-DRA*
3	Antigen processing and presentation	15	5.8 × 10^−5^	*CIITA, HLA-DQB1, HLA-DRB1, HLA-A, HSPA1A, HLA-C, HLA-B, HLA-E, CD74, HSPA6, KIR3DL3, HLA-DPA1, HLA-DPB1, HLA-DOA, HLA-DRA*
4	Phagosome	21	2.0 × 10^−4^	*HLA-DQB1, NOS1, HLA-DRB1, HLA-A, HLA-C, HLA-B, SFTPA1, ITGB3, HLA-E, CLEC4M, FCAR, CD209, COMP, TUBAL3, HLA-DPA1, SCARB1, HLA-DPB1, HLA-DOA, ATP6V0D2, TUBB4A, HLA-DRA*
5	Cell adhesion molecules (CAMs)	19	7.7 × 10^−4^	*PVR, HLA-DQB1, HLA-DRB1, CLDN5, HLA-A, NLGN1, HLA-C, HLA-B, HLA-E, CLDN15, ALCAM, NCAM2, SDC1, CD2, MADCAM1, HLA-DPA1, HLA-DPB1, HLA-DOA, HLA-DRA*

**Table 3:** GEPs DAVID analysis of MDA-MB-231 xenograft mice exposed to 6 Gy of proton irradiation.

Top 5 Molecular Pathways of Differentially Expressed Genes of MDA-MB-231 Xenograft Mice Exposed to 6 Gy of Proton Irradiations				
	Term	Count	*p* Value	Genes
1	Proteoglycans in cancer	22	1.2 × 10^−5^	*NANOG, ERBB4, ROCK2, HCLS1, ERBB2, FASLG, IGF2, FZD3, HGF, DCN, ITGB3, MMP2, PXN, KDR, CTNNB1, SMO, MAPK13, HPSE, PLCG2, HSPB2, PRKACA, TWIST1*
2	Leukocyte transendothelial migration	11	9.5 × 10^−3^	*ITGAL, ROCK2, MAPK13, PLCG2, CLDN5, CTNND1, MYLPF, JAM2, MMP2, PXN, CTNNB1*
3	Graft-versus-host disease	5	3.2 × 10^−2^	*HLA-DQB1, HLA-DRB1, FASLG, HLA-DPA1, HLA-DPB1*
4	Allograft rejection	5	4.6 × 10^−2^	*HLA-DQB1, HLA-DRB1, FASLG, HLA-DPA1, HLA-DPB1*
5	Phagosome	11	4.9 × 10^−2^	*HLA-DQB1, TUBA8, CD36, HLA-DRB1, TUBAL3, HLA-DPA1, SFTPA1, COLEC11, HLA-DPB1, ITGB3, TUBB4A*

**Table 4:** GEPs DAVID analysis of MDA-MB-231 xenograft mice exposed to 9 Gy of proton irradiation.

Top 15 Molecular Pathways of Differentially Expressed Genes of MDA-MB-231 Xenograft Mice Exposed to 9 Gy of Proton Irradiations				
	Term	Count	*p* Value	Genes
1	Proteoglycans in cancer	26	8.4 × 10^−5^	*ERBB2, ITGB3, DCN, MMP2, GPC3, ANK2, HPSE, PPP1R12A, PIK3R5, PIK3R3, PIK3CG, NANOG, WNT10B, ROCK2, ITGA2, IGF2, FZD3, PRKCG, FZD5, FLNB, KDR, WNT2B, EIF4B, MAPK13, VEGFA, WNT11*
2	Rap1 signaling pathway	26	0.0002	*PRKCZ, FGFR3, RAP1GAP, TLN2, CTNND1, LPAR3, FGF13, ITGB3, ITGAM, RAC3, RASGRP2, RAPGEF4, PIK3R5, PIK3R3, ANGPT2, PLCB2, PIK3CG, FYB, GNAO1, GRIN1, PRKCG, KDR, DOCK4, MAPK13, VEGFA, PDGFRA*
3	Pathways in cancer	40	0.0002	*FGFR3, APC2, PTGS2, ERBB2, GNG13, CXCL8, LPAR3, FGF13, MMP2, SUFU, AGTR1, CDKN2A, RAC3, CASP8, RASGRP2, PIK3R5, HHIP, PIK3R3, PLCB2, PIK3CG, CEBPA, PTGER1, COL4A3, WNT10B, HSP90AA1, BCR, VHL, RALBP1, ROCK2, TGFBR2, BRCA2, ITGA2, PRKCG, FZD3, FZD5, STAT1, WNT2B, VEGFA, PDGFRA, WNT11*
4	Signaling pathways regulating pluripotency of stem cells	19	0.0006	*PIK3CG, NANOG, WNT10B, FGFR3, ONECUT1, APC2, PAX6, FZD3, FZD5, WNT2B, RIF1, HAND1, MAPK13, PIK3R5, WNT11, JAK3, SKIL, BMPR1B, PIK3R3*
5	VEGF signaling pathway	11	0.002	*PIK3CG, PTGS2, RAC3, MAPK13, VEGFA, PPP3R2, PRKCG, NOS3, PIK3R5, PIK3R3, KDR*
6	Platelet activation	16	0.005	*PIK3CG, PRKCZ, ROCK2, TLN2, COL3A1, ITGA2, ITGB3, PRKG1, MAPK13, RASGRP2, PPP1R12A, PIK3R5, NOS3, PIK3R3, PLCB2, COL11A1*
7	Phagosome	17	0.008	*HLA-DQB1, NOS1, HLA-DRB1, ITGA2, C1R, SFTPA1, ITGB3, HLA-DQA2, ITGAM, TUBA8, ATP6V0E2, CD209, COMP, TUBAL3, HLA-DPA1, HLA-DPB1, HLA-DRA*
8	Antigen processing and presentation	11	0.008	*HLA-DQB1, HSP90AA1, HSPA2, HLA-DRB1, CD8A, KIR3DL3, HLA-DPA1, HLA-DPB1, HLA-DQA2, CD74, HLA-DRA*
9	Graft-versus-host disease	7	0.008	*HLA-DQB1, CD86, HLA-DRB1, HLA-DPA1, HLA-DPB1, HLA-DQA2, HLA-DRA*
10	Focal adhesion	21	0.009	*PIK3CG, COL4A3, ROCK2, TLN2, ERBB2, COL3A1, ITGA10, ITGA2, PRKCG, ITGB3, FLNB, KDR, RAC3, COMP, VEGFA, PPP1R12A, PDGFRA, PIK3R5, PIK3R3, COL11A1, SHC4*
11	Ras signaling pathway	22	0.01	*PIK3CG, PLD1, PLA2G16, FGFR3, RALBP1, GRIN1, GNG13, PRKCG, FGF13, RGL1, KDR, RAC3, RASGRP2, VEGFA, PDGFRA, ZAP70, PIK3R5, SYNGAP1, PIK3R3, PLA2G2D, ANGPT2, SHC4*
12	Allograft rejection	7	0.01	*HLA-DQB1, CD86, HLA-DRB1, HLA-DPA1, HLA-DPB1, HLA-DQA2, HLA-DRA*
13	Chemokine signaling pathway	18	0.03	*CXCL1, PIK3CG, PRKCZ, FGR, ROCK2, GNG13, CXCL8, CCL4L2, STAT1, CCL15, CCL26, CCR3, RASGRP2, PIK3R5, JAK3, PIK3R3, PLCB2, SHC4*
14	HIF-1 signaling pathway	11	0.04	*PIK3CG, VHL, PFKFB3, HMOX1, ERBB2, VEGFA, PRKCG, NOS3, PIK3R5, PIK3R3, ANGPT2*
15	Wnt signaling pathway	14	0.04	*WNT10B, APC2, ROCK2, PPP3R2, FZD3, PRKCG, FZD5, WNT2B, CSNK2A1, SOST, SFRP1, RAC3, WNT11, PLCB2*

**Table 5:** Gene expression analyses of key genes of TNBC (triple-negative breast cancer) xenograft mice exposed to Proton RadioTherapy PRT.

Gene Expression Analyses of Key Genes of TNBC Xenograft Mice Exposed to PT							
Gene Symbol	Description	2 Gy PRT		6 Gy PRT		9 Gy PRT	
		Array	qRT-PCR	Array	qRT-PCR	Array	qRT-PCR
*BAX*	BCL2 associated X, apoptosis regulator	−1.25	0.51	−1.12	0.32	1.14	2.26
*BCL2*	BCL2 apoptosis regulator	−1.20	0.56	−1.20	0.28	1.18	1.87
*CCNA2*	Cyclin A2	−1.24	0.62	−2.73	0.30	Und.	2.24
*CD24*	CD24 molecule	1.43	1.24	1.23	Und.	Und.	2.92
*CD44*	CD44 molecule	1.37	0.97	1.07	Und.	1.24	3.16
*CDC20*	Cell division cycle 20	−1.11	0.48	−1.11	0.83	1.04	3.83
*CDC25*	Cell division cycle 25	−1.19	0.38	Und	0.51	1.11	1.51
*FOS*	Fos proto-oncogene, AP-1 transcription factor subunit	4.95	3.35	1.12	Und.	1.91	30.56
*FOSB*	FosB proto-oncogene, AP-1 transcription factor subunit	2.51	1.07	1.34	0.57	1.30	6.35
*GDF15*	Growth differentiation factor 15	1.86	1.64	1.72	1.23	2.50	10.27
*MMP9*	Matrix metallopeptidase 9	−1.16	0.21	Und	0.38	1.33	2.31
*RRAD*	Ras related glycolysis inhibitor and calcium channel regulator	1.50	3.78	1.54	1.95	1.71	6.51
*TAF7L*	TATA-box binding protein associated factor 7 like	1.14	1.27	Und	Und.	1.18	1.80
*TNF*	Tumor necrosis factor	1.34	4.14	Und	1.31	1.17	5.02
*TP53*	Tumor protein p53	n.a.	3.11	1.33	1.09	1.25	3.28
*TP53INP1*	Tumor protein p53 inducible nuclear protein 1	1.04	1.40	1.23	1.76	Und.	4.53
*WNT5A*	Wnt family member 5A	1.17	2.12	1.26	2.64	1.14	9.28

Triple negative breast cancer (TNBC); undetected (Und.); proton therapy (PT).

**Table 6:** Top five molecular pathways of differentially expressed genes shared between MDA-MB-231 xenograft mice exposed to 2, 6 and 9 Gy of proton irradiation.

Top 5 Molecular Pathways of Differentially Expressed Genes Shared Between MDA-MB-231 Xenograft Mice Exposed to 2, 6 and 9 Gy of Proton Irradiations				
	Term	Count	*p* Value	Genes
1	Antigen processing and presentation	6	0.003	*HLA-DQB1, HLA-DRB1, KIR3DL3, HLA-DPA1, HLA-DPB1, CD74*
2	Graft-versus-host disease	4	0.009	*HLA-DQB1, HLA-DRB1, HLA-DPA1, HLA-DPB1*
3	Allograft rejection	4	0.01	*HLA-DQB1, HLA-DRB1, HLA-DPA1, HLA-DPB1*
4	Phagosome	6	0.04	*HLA-DQB1, HLA-DRB1, TUBAL3, HLA-DPA1, HLA-DPB1, ITGB3*
5	Complement and coagulation cascades	4	0.05	*F5, CD46, F13A1, C1S*
